# Development of a pVEC peptide-based ribonucleoprotein (RNP) delivery system for genome editing using CRISPR/Cas9 in *Chlamydomonas reinhardtii*

**DOI:** 10.1038/s41598-020-78968-x

**Published:** 2020-12-17

**Authors:** Seongsu Kang, Seungjib Jeon, Seungcheol Kim, Yong Keun Chang, Yeu-Chun Kim

**Affiliations:** Department of Chemical and Biomolecular Engineering, Korea Advanced Institute of Science and Technology (KAIST), Daejeon, 305-701 Republic of Korea

**Keywords:** Gene delivery, Genetic engineering

## Abstract

Recent technical advances related to the CRISPR/Cas9-based genome editing system have enabled sophisticated genome editing in microalgae. Although the demand for research on genome editing in microalgae has increased over time, methodological research has not been established to date for the delivery of a ribonucleoprotein (Cas9/sgRNA complex) using a cell penetrating peptide into microalgal cell lines. Here, we present a ribonucleoprotein delivery system for *Chlamydomonas reinhardtii* mediated by the cell penetrating peptide pVEC (LLIILRRRIRKQAHAHSK) which is in a non-covalent form. Using this technically simple method, the ribonucleoprotein was successfully delivered into *C. reinhardtii*. Gene *Maa7* and *FKB12* were disrupted, and their distinguishing patterns of Indel mutations were analyzed with the observation of several insertions of sequences not originating from the genome DNA, such as chloroplast DNA, into the expected loci. In addition, the cytotoxicity of Cas9 and the ribonucleoprotein was investigated according to the concentration and time in the algal cells. It was observed that Cas9 alone without the sgRNA induces a more severe cytotoxicity compared to the ribonucleoprotein. Our study will not only contribute to algal cell biology and its genetic engineering for further applications involving various organisms but will also provide a deeper understating of the basic science of the CRISPR/Cas9 system.

## Introduction

Clustered regularly interspaced short palindromic repeats (CRISPR)-associated proteins (Cas) have enabled sophisticated genome editing in diverse organisms as a breakthrough methodology^1,2^. Because the CRISPR system was understood as an adaptive immune mechanism in many bacteria and archaea against exogenous DNA fragments in the past decades, many types of CRISPR systems were discovered. Among the various classes and types of CRISPR/Cas systems, class 2 type 2 CRISPR/Cas9 system, in which one RNA-guided endonuclease is required to mediate the cleavage of DNA, has been widely utilized for genome editing for further biological applications^3^. Recently, Cas9 protein has been artificially “renovated” for improved performance by various approaches such as direct evolution^4^. In comparison with other nuclease systems, such as zinc-finger nucleases (ZFNs) and transcription activator-like effector nucleases (TALENs), CRISPR/Cas9 is a simple and efficient tool for genome editing due to its high target specificity and simplicity^2,3^.

CRISPR/Cas9 based genome editing can be achieved by introducing (1) a plasmid vector embedding Cas9-coding gene and a guide RNA(gRNA)-coding gene, (2) ribonucleoprotein (RNP), which consists of Cas9 and a single guide RNA (sgRNA), and (3) mRNA coding Cas9 and gRNA sequences. Among them, ribonucleoprotein delivery has some advantages compared to the others as follows: (1) avoiding random insertional mutagenesis, (2) lower possibility of off-target cleavage, and (3) higher efficiency due to several reasons such as prompt action compared to nucleotide-mediated methods^5,6^. Furthermore, recent studies have shown the RNP delivery method as a promising approach considering that some organisms, especially plants, do not efficiently express the exogenous Cas9 gene, which leads to failure of genome editing based on the CRISPR/Cas9 system^7^.

Microalgae are considered a promising biosource because of their wide range of applications, including biofuel, pharmaceutics, and environmental fields. Algal studies have provided scientific clues for plant science such as the chloroplast, cell cycle, metal transport, etc. by exploiting the homology of genetics and metabolism between microalgae and plants while recognizing that algae experience the evolution of many metabolism pathways of plants^8–10^.

Numerous genetic manipulation strategies of microalgae also have been tried to improve their quality as a bioresource or to contribute basic science. Recent studies revealed that genome editing of microalgae based on the CRISPR/Cas9 system is available and enables sophisticated genetic manipulation^11–20^. Although the significance of genome editing of microalgae based on CRISPR/Cas9 has increased, a methodology for the delivery of effector molecules, especially the ribonucleoprotein, using a cell penetrating peptide has not been established yet. According to previous reports regarding RNP delivery into microalgae for genome editing, many researchers have used only electroporation as a delivery method^11–14^. This appears to be due to the narrow range of intracellular delivery methodologies available for microalgae compared to other organisms and the limited protein delivery efficiency into microalgae^21^. This might be a result of the different physicochemical characteristics of the microalgal cellular barrier, as pointed out in a previous study^22^.

Here, we propose a new methodology for RNP delivery into the microalgae *C. reinhardtii* using the cell penetrating peptide pVEC, which is derived from the murine vascular endothelial cadherin protein. We previously reported a pVEC-mediated protein delivery system into the microalgae cell^23^. As an application of this system, we verified that the RNP can be delivered into *C. reinhardtii* and that the delivered RNP can trigger Indel mutations that are mediated by dsDNA break (DSB) and repair. The *Maa7* gene encoding the tryptophan synthase beta unit and the *FKB 12* gene for peptidyl-prolyl *cis–trans* isomerase were targeted in this study. We observed distinctively different patterns of Indel mutations in the gene disruption of *Maa7* and *FKB12*, respectively; the former showed a strictly controlled triple base-based in-frame shift, and the latter showed a more dynamic and irregular pattern and even a tremendous insertion of DNA that does not mainly originate from gDNA. Most of the inserted DNA was revealed as chloroplast DNA. We also first investigated the cytotoxicity of RNP and Cas9 in the algal cell and observed that the treatment of Cas9 without sgRNA induces a more severe cell cytotoxicity compared to RNP, which may suggest that the sgRNA could act to control or limit Cas9.

Our RNP delivery system based on the pVEC peptide presents the following features: (1) it is not necessary to link the peptide to Cas9 covalently (non-covalent form), (2) time-saving and a simple process (~ 30 min), and (3) cumbersome equipment is not required. This study will contribute to basic algal science as a novel RNP delivery approach for sophisticated genetic manipulation and contribute to other organisms by offering a better understanding of the basic science of CRISPR/Cas9.

## Results and discussion

### Translocation of Cas9/sgRNA ribonucleoprotein by pVEC into* C. reinhardtii*

There have been a few studies on the delivery of the ribonucleoprotein into cells for genome editing using cell penetrating peptides in the following manners: (a) Conjugation of the cell penetrating peptide to Cas9 by genetic engineering^24^ and (b) non-chemical conjugation^25^. When delivering the ribonucleoprotein using the cell penetrating peptide-conjugated Cas9, R9 conjugated Cas9 can penetrate mammalian cell lines leading to gene disruption with reduced off-target mutations^24^. There have been some studies on the delivery of Cas9 without conjugation with CPP, also targeting mammalian cell lines. As mentioned in previous reports, studies on molecular manipulations, especially using cell penetrating peptides, for microalgae have been limited and are not fully understood compared to mammalian cell lines^22,23^.

**Figure 1:**
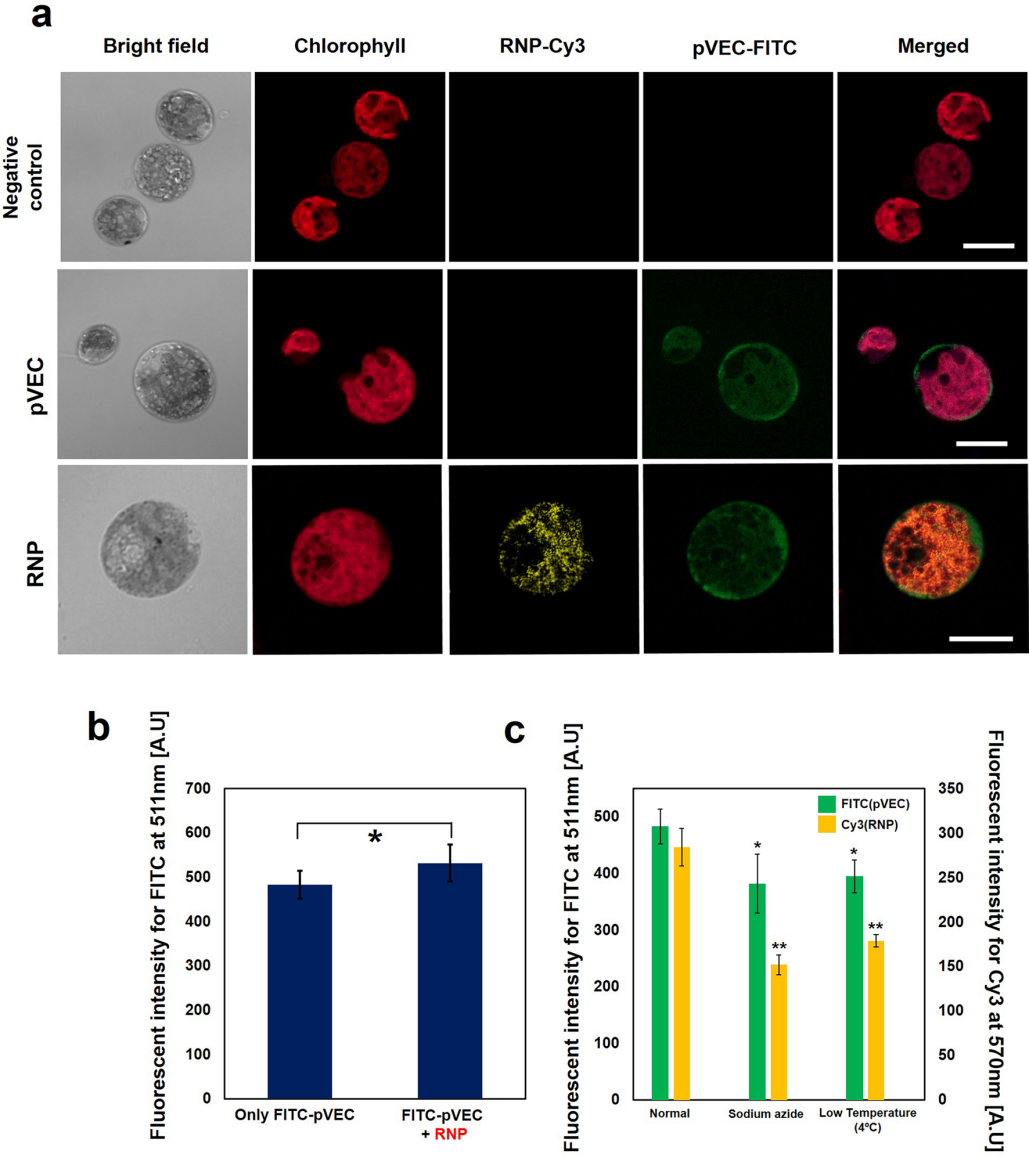
Translocation of cell penetrating peptide pVEC and ribonucleoprotein. (**a**) The confocal microscopy image for *C. reinhardtii* to investigate the translocation of pVEC (FITC) and RNP(Cy3). Cas9 and sgRNA were incubated for 30 min before treatment, and mixed with a cell sample. pVEC was added to the cell sample containing RNP and incubated for 20 min. After washing including trypsin treatment, cells were observed under confocal microscopy. White scale bar represent 10 μm. (**b**) Translocation of pVEC-FITC. The washed sample was resuspended in 200ul of TAP media and its fluorescent intensity at 511 nm was measured by a spectrofluorophotometer with excitation wavelength of 475 nm (**c**) Treatment of endocytosis inhibitor. The fluorescent intensity was measured at 511 nm and 570 nm for FITC and Cy3 respectively. Sodium azide (SA) was treated for 1 h with the final concentration of 10 mM. The data represent the average of n = 3 replicate experiments. Standard deviation bars are shown. **Significantly different (Student’s t-test, *p* < 0.05) and *Significantly not different (Student’s t-test, *p* > 0.05).

In our previous study on protein delivery systems, we verified that the 18-length peptide pVEC (LLIILRRRIRKQAHAHSK) from murine vascular endothelial cadherin mediates the delivery of various kinds of proteins into microalgae cell lines in a non-conjugation form. We hypothesized that the cell penetrating peptide pVEC could deliver the Cas9/sgRNA complex ribonucleoprotein (RNP) into *C. reinhardtii*. To identify the translocation of the ribonucleoprotein into the *C. reinhardtii* cell, cy3-conjugated Cas9 and FITC- conjugated pVEC were used in this study. As seen in Fig. 1a, FITC-conjugated pVEC and Cy3-conjugated RNP (precisely, Cy3 conjugated Cas9) were delivered into *C. reinhardtii*. After treatment, the Cy3 signal was detected in the cell body evenly, which is a similar trend of protein delivery by pVEC documented in our previous study^23^. It was also observed that the cells with Cy3-RNP in their cell bodies also had a high level of FITC signal from FITC-pVEC while there were also many cells with a FITC signal but without a Cy3 signal, which implies that only pVEC and not RNP was translocated into the cell (data not shown). FITC-conjugated pVEC penetrated into *C. reinhardtii* in both the pVEC only treated group and pVEC and RNP-treated group without a significant difference in the fluorescent intensity of FITC (Fig. 1b). In addition, the Cy3-RNP was monitored after 18 h of treatment. (Figure S1) it was observed that the Cy3 signal moved to the chlorophyll signal(red)-free zone which indicates cytosol and nucleus region according to the previous research. Further investigation seems to be necessary for the clear answer.

We investigated whether either pathway is related to the delivery of RNP into the cell. Our previous study^23^ revealed that pVEC delivered the exogenous protein into the algal cell through both energy-dependent endocytosis and direct penetration. The fluorescent intensity of FITC at 511 nm and the intensity of Cy3 at 570 nm were measured simultaneously under the treatment of an endocytosis inhibitor, sodium azide, which showed the most significant inhibition effect on protein uptake in a previous study, and low temperature (4ºC) (Fig. 1c). The fluorescent intensity of FITC decreased by 20% under the treatment of sodium azide, while the fluorescent intensity of Cy3 decreased by 46%. This observation implied that the cell penetrating peptide pVEC is less affected by the endocytosis inhibitor, while intracellular delivery of the protein by the peptide is strongly affected. The dosage effects of sodium azide on the uptake of pVEC and the delivery of protein by pVEC into the cells were investigated, respectively, in Figure S2. Collectively, it can be concluded that the direct pathway is more dominant for the translocation of the cell penetrating peptide than for the delivery of proteins, and endocytosis is more dominant for protein delivery than for translocation of the cell penetrating peptide. This observation is coincident with a previous mechanism study regarding the translocation of pVEC, showing that direct penetration is strongly related to the pVEC^26^. In addition, our observation that different mechanisms govern the penetration of the cell penetrating peptide and the delivery of the protein, respectively, when using the same peptide provides new insight for understanding the mechanism of the cell penetrating peptide, which has remained elusive to date.

**Figure 2:**
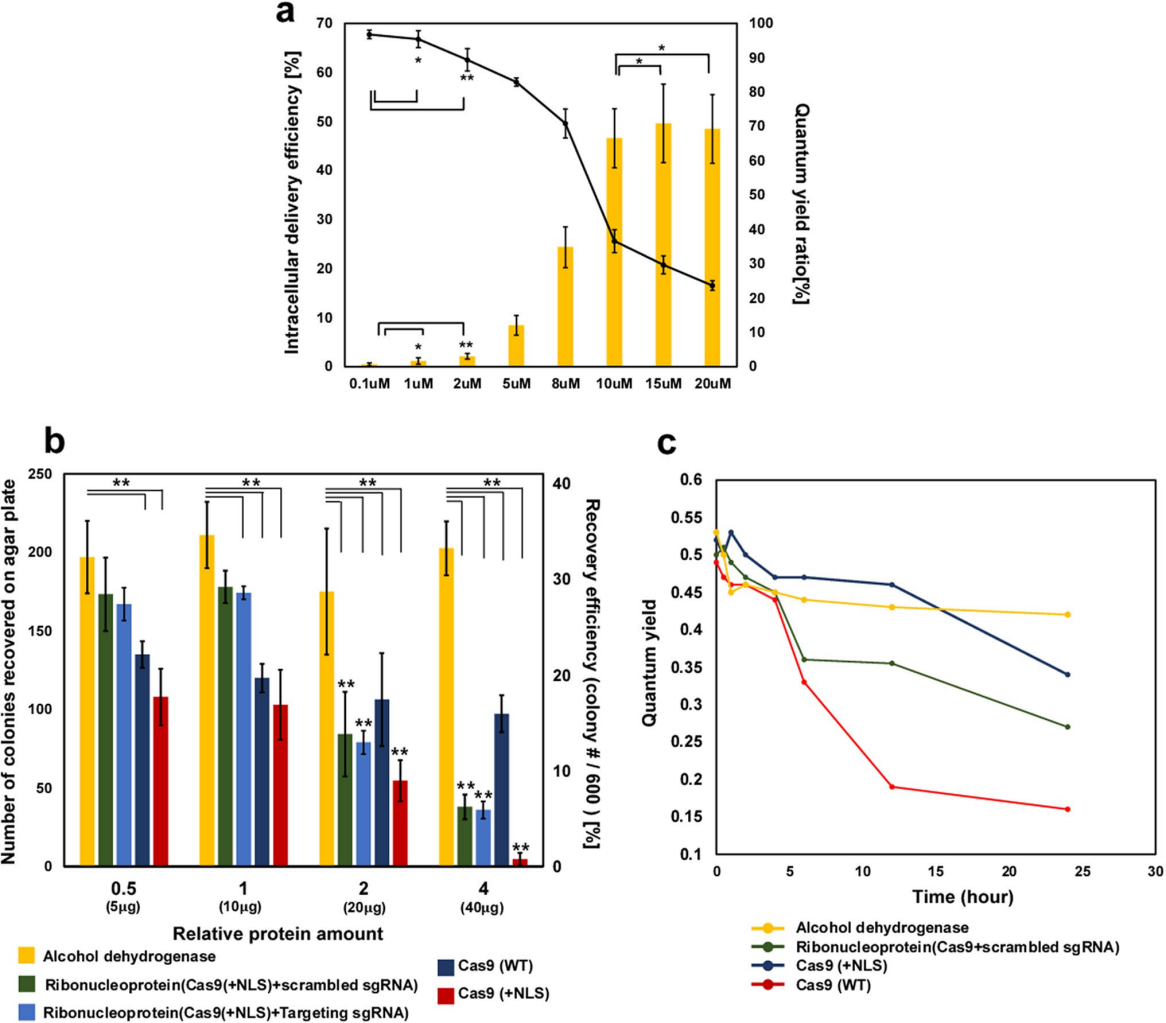
Delivery efficiency of RNP and Cytotoxicity of Cas9 and ribonucleoprotein. (**a**) The cellular uptake efficiency of Cy3-RNP into wild-type *C. reinhardtii* measured by a flow cytometer according to the concentration of pVEC. The ratio of the quantum yield of wild-type *C. reinhardtii* according to the concentration of pVEC. After incubation of samples for one hour under dark as a dark adaptation, quantum yield was measured by AquaPen-C APA100. The ratio of the quantum yield was calculated by dividing the QY of the experimental group by the QY of the control group. The data represent the average of n = 3 replicate experiments. Standard deviation bars are shown. **Significantly different (Student’s t-test, *p* < 0.05). *Significantly not different (Student’s t-test, *p* > 0.05). (**b**) The recovery of algal cell on agar plate after treatment. Relative protein amount 1 represents 10ug of protein (Cas9 and alcohol dehydrogenase). 600 cells were spread and counted. The final concentration of pVEC for delivery of protein was 8uM. **Significantly different (Student’s t-test, *p* < 0.05). (**c**) The quantum yield for algal cell depends on time. Relative amount 1 of protein was used. The quantum yield was measured by AquaPen-C APA100. The data represent the average of n = 3 replicate experiments. Standard deviation bars are not shown for legibility.

We also investigated the relationship between the concentration of pVEC and the delivery efficiency of RNP into the cells (Fig. 2a). The protein delivery by pVEC becomes significant over 2–5 μM of pVEC, which is a similar trend to a previous study^23^. The delivery efficiencies of RNP into wild-*type C. reinhardtii* were 8.41% for 5 μM of pVEC, 24.36% for 8 μM of pVEC, and 46.56% for 10 μM of pVEC. At over 10 μM of pVEC as a final concentration in the treatment, the delivery efficiency of RNP reached a plateau of about 45%. Otherwise, the quantum yield ratio decreased when the concentration of pVEC was increased further and especially showed a dramatic decrease in a range of 8–10 μM. In the subsequent experiments for genome editing, 8 μM of pVEC was used.

### The effect of the ribonucleoprotein on algal cell viability

Because genome editing based on the CRISPR/Cas9 system emerged as a powerful genetic manipulation tool, it was steadily suggested that Cas9 expressed from an exogenous gene or Cas9 delivered from the extracellular environment may trigger cell death due to their toxic effects^18,27,28^. The adverse effect on cell viability due to the unknown toxic effects is considered one of the hurdles to overcome for future applications, which has led to research on improving the on-target efficiency by mutagenesis^4^.

Although the potential toxicity of Cas9 in *Chlamydomonas* has been noted earlier^18^, this phenomenon is not fully understood and has not been studied before. Here, we investigated the viability of algal cells using the following proteins: wild type Cas9 deficient in NLS, Cas9 with an NLS tag (Cas9-NLS), RNPs, and alcohol dehydrogenase as a control which has a similar molecular weight as Cas9 of 150 kDa (Fig. 2b). In a previous study, we observed that alcohol dehydrogenase is delivered into *C. reinhardtii* by pVEC. The same molar concentration was used for all the proteins in the treatments. Relative amount 1 refers to 10 μg of protein for 3 × 10^7^ cells while relative amount 2 and 4 refer to 20 and 40 μg of protein for 3 × 10^7^ cells. Relative amount 0.5 refers to 5 μg of protein for 3 × 10^7^ cells. For the RNP group, sgRNA, which targets the *Maa7* locus (demonstrated later), was used. Cells transfected with proteins were spread onto TAP agar plates after 15 h of treatment. The difference in molecular uptake for Cas9 and RNP by pVEC was not significant (Figure S3).

When the relative amount of protein was increased by showing about 40%, the cell viability for the alcohol dehydrogenase-delivered cells did not show a significant decrease. Meanwhile, the cell viability for the wild-type Cas9, Cas9-NLS, and RNP groups showed a steady decrease with relative amount 2. The insignificant difference in the cell viability when the concentration of alcohol dehydrogenase increased implies that the loaded amount of protein in the cell does not affect the cell viability.

The group for the ribonucleoprotein that comprises Cas9-NLS and sgRNA targeting the *Maa7* gene site showed a higher cell viability compared to the groups treated with Cas9-NLS without sgRNA, with a cell viability of 34.66% for relative amount 0.5, 35.6% for relative amount 1, 16.86% for relative amount 2, and just 7.6% for relative amount 4 (light blue bar in Fig. 2b). It was observed that there was no significant difference between the groups for the scrambled sgRNA and targeting sgRNA. This observation suggests that a higher concentration of RNP could not give a higher number of mutant cells due to a dramatic decrease in viability at a high concentration of RNP. We could not obtain any 5-fluoroindole-resistant mutants (see details below) for relative amount 4 (Cas9 40 μg and sgRNA 53.2 μg per 3 × 10^7^ cells) of RNP.

Interestingly, the most distinguishing feature is that the Cas9-NLS treatment alone (red bar in Fig. 2b) resulted in the lowest viability, 21.6% for relative amount 0.5, 20.6% for relative amount 1, 10.92% for relative amount 2, and just 0.92% for relative amount 4. It can be concluded that solo Cas9 without sgRNA induces a more severe cell cytotoxicity compared to the ribonucleoprotein. This observation is quite similar to a previous study regarding the cell cytotoxicity of a zinc finger^29^. At the same time, it is slightly different (or conflicts with) to another study which reported the viability of primary mouse T cells electroporated with a high concentration of Cas9 exceeding 80%^6^. Based on our knowledge and previous studies, it can be surmised that the cytotoxicity of Cas9 causes a significant decrease in the cell viability of algal cells. The higher viability for wild-type Cas9 deficient in NLS (dark blue bar in Fig. 2b) supported this conclusion and suggests that the cytotoxicity can be attributed to the nuclease activity of Cas9 in the nucleus, considering that the NLS sequence mediates the efficient access of the nuclease to the genome in the nucleus. Based on previous studies, off-target cleavage of the genome by Cas9 appears to cause severe cytotoxicity in *C. reinhardtii*^18,19^, which is also similar to the observation that Cas9 causes cytotoxicity in mammalian cells^30,31^. Our observation is similar to a previous finding that Cas9 results in severe cytotoxicity on specific organisms, such as *Schizosaccharomyces pombe*^32^. The question of whether CRISPR/Cas9 is not “favorable” to microalgae cell lines as it is to zebra fish^33^ and Xenopus^34^, which are known to be ineffective with the CRISPR/Cas9 system, has remained unsolved and should be investigated in further studies.

The decrease in the cell viability of various organisms when using wild-type Cas9 appears to be due to its possibility to access the genome by nucleus transport pathways or by other opportunities such as cell division.

Considering the series of past observations, sgRNA itself appears to have an important role in suppressing robust off-targeting cleavage of the genome by Cas9. Previous research also observed RNA-independent DNA cleavage activities of Cas9 in the presence of Mn^2+^ ions^35^. It is noteworthy that manganese is essential for the photosynthesis system of algae and included in TAP media which we used in our research^36^. For a more detailed and apparent investigation and verification, analysis of the whole sequence for detecting off-targeting of the CRISPR/Cas9 system based on NGS-based deep sequencing appears to be necessary to investigate how Cas9 affects cell viability. It seems that an investigation using “dead” Cas9, which has inactivated nuclease sites, may offer a deeper understanding of this phenomenon in future work.

We measured the quantum yield of cell samples to investigate how intracellularly delivered protein affects the cell viability of algal cells according to time (Fig. 2c). Although the quantum yield just indirectly estimates the algal cell viability, it has been used as a useful method due to its promptness of measurement. Our group also reported that there is a highly close correlation between the quantum yield and the cell viability of an algal cell on an agar plate in a previous study^23^. All experimental groups showed no significant difference until 4 h after treatment. The quantum yield for the RNP group and Cas9-NLS group dramatically decreased after 6 h. Based on previous studies regarding Cas9 enzymatic kinetics on time^37^, it appears that the nuclease activity of Cas9 induced a phenotypic change in the cells at that time. The group for wild type Cas9 showed a dramatic decrease that was delayed compared to the other groups at 12 h.

### Gene disruption of Maa7 in *C. reinhardtii* and its subspecies

**Figure 3:**
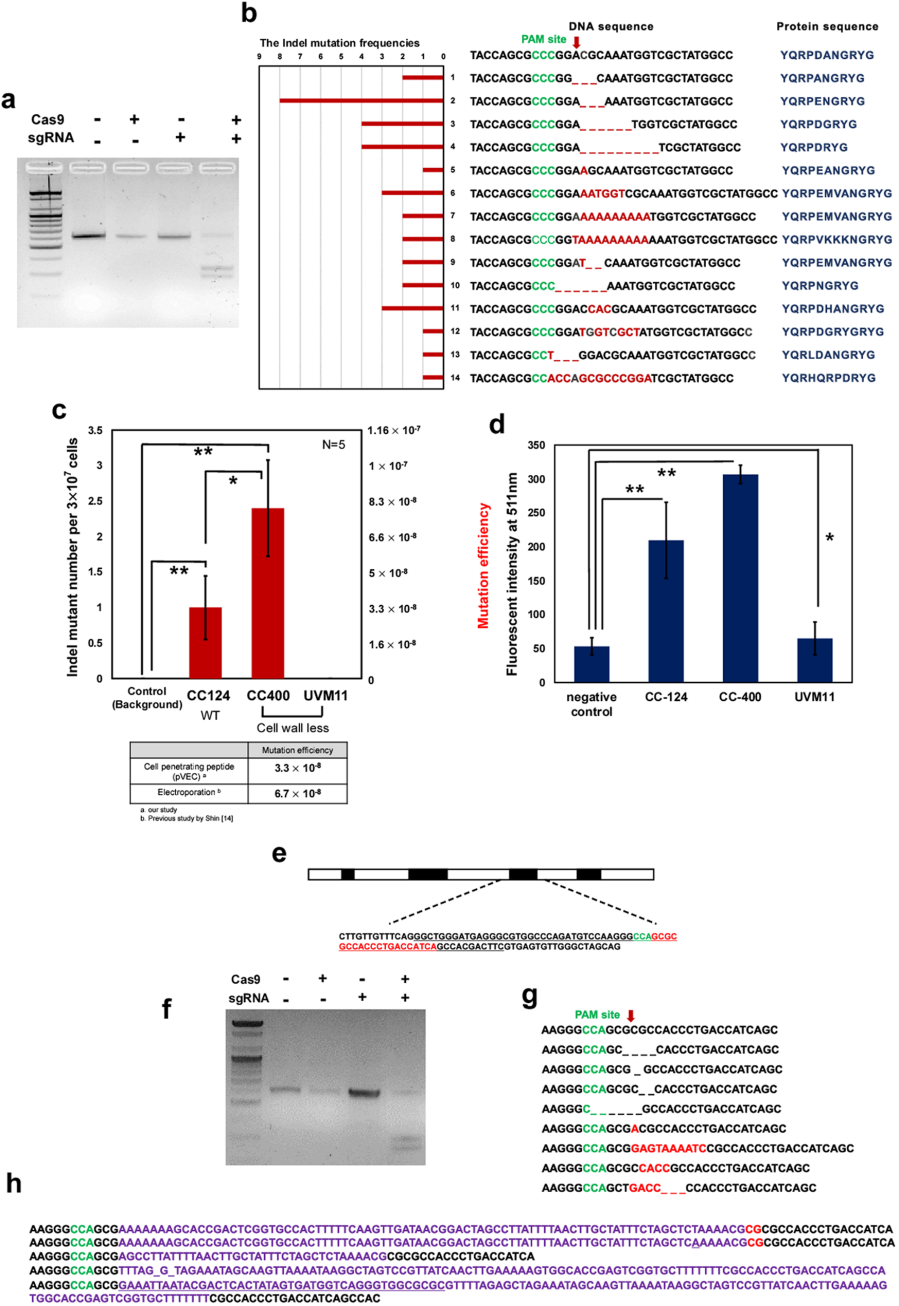
Gene disruption of Maa7 responsible for tryptophan synthase beta unit (TSB) and gene disruption of FKB12. (**a**) In-vitro cleavage assay. PCR amplicon having a size of ~ 600 bp was cleaved into two fragments having size of 350 bp and 250 bp. Template DNA of 100 ng was used for the assay and 600 ng of Cas9 and 500 ng of sgRNA were used for in-vitro cleavage assay. Detailed methodology was described in the method part. (**b**) The Indel mutation cases for in-vivo genome editing experiments. The PAM site is shown in green, and the cleavage point by sgRNA is shown as a red arrow. (**c**) Indel mutation efficiency of *C. reinhardtii* subspecies CC124, cell wall-less mutant CC400 and UVM11. For cell wall-less mutant, a starch-embedding method was used for spreading. (**d**) Protein(FITC-BSA) delivery into *C. reinhardtii* subspecies by 8 μM of pVEC. Fluorescent intensity of FITC at 511 nm was measured. The washed sample was resuspended in 200ul of TAP media and its fluorescent intensity at 511 nm was measured by a spectrofluorophotometer with excitation wavelength of 475 nm. (**e**) Description of FKB 12 locus. Underlined sequences refer to CDS, green-sequence refer PAM site, and red-sequence refer sgRNA target site. (**f**) In-vitro cleavage assay using PCR amplicon of 600 bp. (**g**) Indel mutation cases for in-vivo genome editing experiments. (**h**) Insertion of in-vitro transcription DNA template. The data represent the average of n = 5 replicate experiments. Standard deviation bars are shown. **Significantly different (Student’s t-test, *p* < 0.05) and *Significantly not different (Student’s t-test, *p* > 0.05).

Referencing a previous study on *Maa7* gene disruption in *C. reinhardtii*^14^, we synthesized sgRNA by in-vitro transcription using T7 RNA polymerase. Figure 3a shows that RNP can cleave the PCR amplicon of *Maa7* with 600 bp size into two fragments with a size of 250 and 350 bp in an in-vitro cleavage assay.

The mutation of *Maa7* results in resistance against 5-fluoroindole in the media. In the in-vivo cell experiment for gene disruption, various types of Indel mutations were observed including insertions, deletions, and a combination of insertion and deletion (Fig. 3b). In the analysis of all Indel mutations that we achieved in the series of experiments, the majority of Indel mutations occurred at the expected cleavage site (Red arrow). The triple base deletion at the expected cleavage site occurred with the highest frequency of 23.5%. Although genome editing mediated with CRISPR/Cas9 ensures specificity by the sgRNA complementary to a target sequence, it was observed that RNP induced Indel mutations in the surrounding sequences from the expected cleavage point (red arrow) and even disrupted the PAM site, as described in cases 13 and 14. It was also observed that the deletion, insertion, or substitution of 3 base pairs was dominant in the majority of the mutations, referring to an in-frame mutation, similar to a previous report^14^. As seen in the peptide sequence, none of the Indel mutations affected the later sequence of amino acids after arginine (R). This implies that the RPDAN motif of the tryptophan synthase beta unit (TSB) is involved in not only enzymatic function but also other critical functions in organisms, as suggested in a previous report^14^.

Interestingly, it was observed that some poly A was inserted in the cleavage site, as seen in cases 7 and 8. Although a recent study concluded that more than 6 bp DNA fragments from the template DNA for in-vitro transcription were inserted into the cleavage site^7^, it is not clear whether the poly A sequences in our observation come from the in-vitro transcription template. In the sequencing analysis, we did not find any off-target mutations in the target, the *Maa7* locus, and did not obtain any Indel mutations in the negative control that was treated with the pVEC without RNP.

We also attempted RNP delivery into *C. reinhardtii* sub-species, CC400 and UVM 11, which both are mutants without cell walls (Fig. 3c). The mutant CC400 showed a higher mutation efficiency of 8 × 10^–8^ compared to the wild type CC-124 at 3.3 × 10^−8^, which is 2.44-fold higher. This result showing the effect of the cell wall on RNP delivery efficiency is similar to a previous study that showed more successful gene disruption in the mutant CC-1883 without a cell wall can be achieved compared to wild-type *C. reinhardtii* when RNP is delivered by electroporation^17^. It was also shown that the mutation efficiency of wild type using pVEC is 3.3 × 10^−8^, which is almost half of the mutation efficiency of 6.7 × 10^−8^ that Shin’s previous study showed. In Shin’s previous study, the RNP was delivered by electroporation, and the same gene *Maa7* was targeted in wild type *C. reinhardtii*.

Interestingly, UVM11, which is mutant without a cell wall generated by UV mutagenesis for a high expression level of transgenic protein, did not result in a gene disruption mutant. The reason for the failure of a gene disruption in the UVM11 cell line through RNP delivery mediated by pVEC appears to be that the protein delivery by pVEC is not available in the UVM cell line, as described in Fig. 3d which covers the experimental data for protein delivery by pVEC. It was observed that the electroporation of UVM11 with RNP resulted in some mutants with lower mutation efficiency (~ 1.5 × 10^−8^) and weak statistical significance (*p* = 0.051) compared to the negative control and pVEC-treated group. (Figure S4) It is not clear why UVM 11 shows the lower Indel mutation efficiency compared to wild-type. Further investigation whether UVM11 strain is not favorable to CRISPR/Cas9 system, or vulnerable to the disruption of Maa7 seems to be required for this question.

### Gene disruption of FKB12

Previous studies showed that the disruption of the peptidyl-prolyl *cis–trans* isomerase gene (*FKB12* gene) renders an increase of resistance against rapamycin in *C. reinhardtii*^17,38^. The third CDS region (69 base pairs) of *FKB12* was targeted as described in Fig. 3e, and the sgRNA was synthesized by a previously mentioned method, and we checked whether the RNP could cleave the PCR amplicon for *FKB 12* in-vitro (Fig. 3f). Interestingly, numerous types of mutations were observed (Fig. 3g, h, and Table 1). Unlike the Indel mutations from the disruption of *Maa7*, not only insertion or deletion mutations based on triple bases but also insertions or deletions of various numbers of bases were observed. Compared to the gene disruption of *Maa7* by RNP, it was observed that DNA base pairs longer than 10 bp were inserted into the cleavage point. Some of the inserted sequences were revealed as the DNA template for in-vitro transcription and other endogenous DNA sequences. As seen in Fig. 3h, part of the DNA template for the in-vitro transcription or the whole DNA template was inserted into the cleavage point. This observation is coincident with a previous report in which researchers observed the insertion of the DNA template for in-vitro transcription despite Dnase treatment during the purification of sgRNA^7^.

**Table 1:**
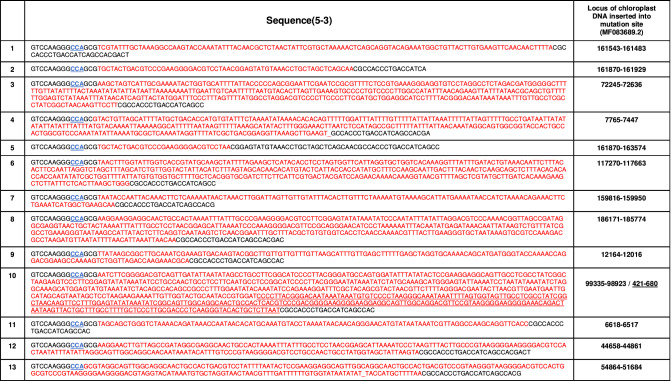
Mutations with insertion of chloroplast DNA in FKB 12 gene disruption.

Based on chloroplast DNA sequence MF083689.2, inserted DNA was demonstrated in red. PAM site was demonstrated in blue with underline.

One of the distinguishing features of the insertion of the long DNA sequence was that partial DNAs from the chloroplast with a size of 60–600 bp were inserted into the cleavage point precisely, which has not been reported before (Table 1). Considering that no donor DNA homologous to the DNA sequences of the target site was supplied, it appears that the insertion was mediated by non-homologous end joining (NHEJ). However, it is not clear whether a fragment of chloroplast DNA was generated by fragmentation (or degradation) induced by an exogenous source triggered by cell damage during transfection or generated by an endogenous mechanism. Previous studies revealed that the DNA of cell organelles such as mitochondria and chloroplast might suffer degradation by various sources such as reactive oxygen species (ROS) and lysosomes^39,40^. We could not rule out the possibility that the chloroplast DNA may be fragmented during the pVEC treatment, which triggers severe cytotoxic stress, or that lysosomes released during the destruction of the chloroplast may degrade the chloroplast DNA.

Previous studies have shown frequent gene transfer from the chloroplast genome to the nucleus^41^. Furthermore, the escape of organelle DNA and its uptake into the nucleus has been shown experimentally, and it was shown that NHEJ might highly contribute to this phenomenon^42,43^. Additionally, a previous study also revealed that the CRISPR/Cas9 system could result in large insertions in the NHEJ process such as 140, 216, and 448 bp, which suggests insertion of DNA of more than 100 bp is not impossible in CRISPR/Cas9 cleavage and NHEJ repair^44^. A more detailed investigation is necessary to verify whether double-strand DNA breaks artificially induced by the CRISPR/Cas9 system “promote” the recombination of chloroplast DNA into a nucleus genome.

We achieved 13 mutations with greater than 99% coincidence with chloroplast DNA (MF083689.2). In the case of mutation 10 in Table 1, two fragments of chloroplast, 99,335–98,923 and 421–680, were sequentially inserted into the cleavage point. Compared to the gene disruption of *Maa7*, the gene disruption of *FKB12* permitted diverse Indel mutations, which implies that the mutation of *FKB12* is not critical to cell survival, unlike *Maa7*. FKBP12 is known to have the peptidyl prolyl cis/trans isomerase activity that is involved in protein-folding and identified in bacteria, fungi, animals, plants, and *C. reinhardtii*^38^. Previous studies have shown that FKBP12 interacts with the TGF-β receptor and the Ca^2+^ releasing ryanodine receptor^45,46^ as an important immunophilin, and its knockout mouse models exhibit embryonic lethality, hyperinsulinemia, and cardiac defects^47,48^. On the other hand, its role or function in non-mammalian species is not well understood. Loss of FKBP12 function in *Saccharomyces cerevisiae* and *C. reinhardtii* does not affect the viability^38,49^. This characteristic of FKB12, not essential to cell survival, seems to enable various types of insertions into the cleavage point.

Similarly, some mutations in which in-vitro transcription DNA template and endogenous coding sequence were inserted sequentially were observed (Figure S5). In a series of experiments, the mutation efficiency of 1.1 × 10^–6^ was achieved in the gene disruption of *FKB12*.

## Conclusion

Although genetic engineering of microalgae is urgently required for further applications of microalgae in industry, efficient and precise genome editing based on the CRISPR/Cas9 system into microalgae has not been fully established, in contrast to other organisms. The demand for methodological studies on the delivery of effector molecules for CRISPR/Cas9, such as RNP, also has increased. In this study, we propose a new methodology for the delivery of Cas9/sgRNA RNP into *C. reinhardtii* using the cell penetrating peptide pVEC in a non-conjugation form. This RNP delivery system is simple and cost-effective and does not require any expensive or cumbersome equipment such as a pulse generator, and it is not necessary to link the peptide to Cas9 by chemical covalent linking. Using our delivery system, gene disruptions of *Maa7* and *FKB12* were successfully achieved, offering a broad analysis of Indel mutations by CRISPR/Cas9 in microalgae, with numerous cases compared to previous reports. Furthermore, some particularly unusual mutations in which chloroplast DNA is inserted into the expected cleavage point were observed, which has not been reported before. We investigated the cytotoxicity of Cas9 and RNP on algal cells as the first report and revealed that treatment with Cas9 without sgRNA showed a higher cytotoxicity than the treatment with RNP. This may suggest a mitigation effect of sgRNA on Cas9′s off-target action inducing cytotoxicity, which also suggests room for further investigation. This study will greatly contribute to algal studies and trigger active and broad research regarding genome editing based on CRISPR/Cas9 as a novel methodology. Besides the contribution to algal studies, our experimental observations on the cytotoxicity of Cas9 and RNP and the distinctive insertional or deletion mutations may offer insight for a better understanding of the science of CRISPR/Cas9.

## Methods

### Cell preparation

All *C. reinhardtii*, wild type (CC-124), and mutants without cell walls, CC-400 and UVM11, were cultured in tris–acetate-phosphate(TAP) media with continuous illumination, constant temperature (25 °C and agitation (140 rpm). UVM11 strain was kindly provided by Dr. Ralph Bock through legitimate MTA. The recipe and composition for TAP media followed the guidelines of the *Chlamydomonas* resource center and report^50^. At the early exponential phase, the cells were harvested using centrifugation and washed using MAX Efficiency Transformation Reagent for Algae (A24229, ThermoFisher Scientific, USA) and resuspended in that buffer with a final concentration of 3 × 10^8^ cells/ml. No significant difference between TAP and MAX Efficiency Transformation Reagent was observed in terms of the delivery efficiency of the protein and the genome editing.

In the harvesting step, cells should not exceed a density of 3 × 10^6^ cells/ml because it was observed that over-grown cells could result in even a lower mutation efficiency.

### Preparation of ribonucleoprotein

Both wild-type Cas9 without NLS (Cas9 Nuclease, M0386M, NEB, US) and Cas9 with NLS (EnGen Cas9 NLS, M0646M, NEB, US) proteins were purchased from New England BioLabs. sgRNA was synthesized using T7 in-vitro transcription according to the protocol provided by the manufacturer (NEB). Briefly, the DNA template for in-vitro transcription was amplified using Q5 High-Fidelity DNA polymerase (M0491M, NEB, US) with the following protocol: 98 °C for 1 min, 98 °C for 10 s for denaturation, 54 °C for 15 s for renaturation, 72 °C for 20 s for extension, and 72 °C for 5 min for final extension. PCR cycles were repeated 25–35 times. Using the purified PCR amplicon, the sgRNA was synthesized using T7 RNA polymerase and other ingredients mentioned in the manual overnight at 37 °C. Synthesized RNA was purified using the conventional nucleic acid purification kit (28104, Qiagen, German). Primers used for the experiments are described in the supplementary data.

### In-vitro cleavage assay for the target DNAs with the ribonucleoprotein

In-vitro cleavage assay verified the activity of the ribonucleoprotein, according to a previous paper^14^. Briefly, 500 ng of sgRNA and 600 ng of Cas9 protein were incubated at room temperature for 15 min. 100 ng of PCR amplicon with the target cleavage site (650 bp) was mixed with 1 μl of NEB 3.1 buffer in a final volume of 10 μl. After 2 h of reaction at 37 °C, 1 μl of RNaseA (KB-0101, Bioneer, Korea) was added and incubated for 20 min. The incubated mixture was purified using the conventional nucleic acid purification kit, and the final product was loaded onto a 2% agarose gel for electrophoresis.

### Transfection of the ribonucleoprotein by the cell penetrating peptide pVEC

Referring to a previous study^14^, 10 μg of Cas9 and 13.3 μg of sgRNA were incubated at room temperature for 15 min. A prepared cell sample of 100 μl at 3 × 10^8^ cells/ml was added to the incubated ribonucleoprotein and gently mixed by fingertip or pipette. The cell-penetrating peptide pVEC (LLIILRRRIRKQAHAHSK, manufactured by Peptron Inc, Korea) was added to the sample and mixed immediately. In a series of experiments, we observed that there was no significant difference between the two types of treatment with pVEC: (1) pre-incubation of RNP and pVEC for the formation of complex, and (2) treatment of pVEC and RNP to the cell simultaneously. Based on the price of the pVEC offered by Peptron, it costs $ 0.15 per sample (3 × 10^7^ cells).

After incubation of the cells mixed with the RNP and pVEC for 30 min at 25 °C, trypsin was added, and the mixture was incubated for 15 min at 37 °C. The sample was washed by TAP media and transferred to 10 ml of TAP media and incubated for 15 h under dim light without shaking as a “recovery” step. For the *Maa7* gene disruption experiments, cells were transferred to 10 ml of TAP media containing 1.5 mM of L-tryptophan. Recovered cells were spread on 1.5% TAP agar containing 1.5 mM of L-tryptophan and 35 μM 5-fluoroindole for the gene disruption of *Maa7*. After 2 weeks, mutant cells were selected, and Indel mutations were analyzed using colony PCR.

For gene disruption of peptidyl-prolyl *cis–trans* isomerase gene (*FKB12* gene; 12-kDa FK506-binding protein), which mediates the interaction between rapamycin and Target of Rapamycin (TOR), recovered cells under dim light were transferred to 50 ml of TAP media containing 500 nM of rapamycin and incubated for 7 days at 25 °C with agitation (140 rpm). Incubated cells were then spread onto 1.5% TAP agar plates containing 20 μM rapamycin. The reason why we incubated the cells in liquid media containing rapamycin is that rapamycin could not effectively select mutant cells perfectly while numerous false positive colonies emerged after 10 days. The high frequency of false positive colonies in the selection appears to be due to rapamycin not being able to block the cell cycle permanently, which is similar to a previous report that the inhibition effect of rapamycin on *Chlamydomonas* proliferation diminished when time passed^38^.

After the transfection of the mutant without a cell wall, the cells were spread on starch-embedded agar plates, as described in a previous paper^44^.

### Analysis of the delivery efficiency and viability

Localization of pVEC and Cas9 was observed using confocal laser scanning microscopy (FV1000 Live, Olympus, Japan). Peptron Inc. also prepared the fluorescein-5-isothiocyanate (FITC)-conjugated pVEC. Cy3-conjugated Cas9 was purchased from ToolGen, Inc (Korea). As mentioned above, the trypsin was treated before the microscopic analysis because pVEC induces strong interaction between foreign protein and the cell surface even though foreign protein is not actually delivered into the cell^23^. The delivery efficiency was measured by a cell cytometer (FACS Calibur, BD Biosciences, USA), and the cell was regarded as “fluorescing” when its fluorescence intensity was higher than the background signal from 99% of the untreated control cells. The quantum yield for estimating the cell viability was measured using an AquaPen-C APA100 (Photon System Instruments, Czech Republic). Before measuring the quantum yield, the sample was incubated in the dark for one hour as a dark adaptation.

## Supplementary information

Supplementary Information 1.
