# Supplementary material for: Development of a pVEC peptide-based ribonucleoprotein (RNP) delivery system for genome editing using CRISPR/Cas9 in Chlamydomonas reinhardtii
Source: Sci Rep. 2020 Dec 17;10:22158. doi: 10.1038/s41598-020-78968-x (PMC7747696; doi:10.1038/s41598-020-78968-x)
Supplement: Supplementary file 1 — Supplementary Information 1. [file 41598_2020_78968_MOESM1_ESM.docx]

**Supplementary Information**

**Development of a pVEC peptide-based ribonucleoprotein (RNP) delivery system for genome editing using CRISPR/Cas9 in *Chlamydomonas reinhardtii***

**Seongsu Kang^1^, Seungjib Jeon^1^, Seungcheol Kim^1^, Yong Keun Chang^1^, and Yeu-Chun Kim^1*^**

^1^ Department of Chemical and Biomolecular Engineering, Korea advanced Institute of Science and Technology (KAIST), Daejeon 305-701, Republic of Korea

^*^ Corresponding Author: dohnanyi@kaist.ac.kr

The author(s) responsible for distribution of materials integral to the findings presented in this article in accordance with the policy described in the Instructions for Authors is : Yeu-Chun Kim (dohnanyi@kaist.ac.kr)

**
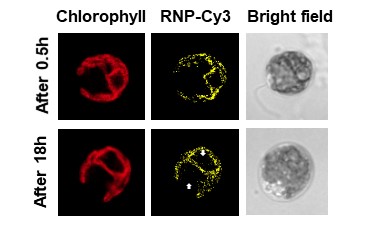
**

**Figure S1. Monitoring the RNP-Cy3 after 18 hours of pVEC treatment.**

White arrow indicates the Cy3 signal detected in the chlorophyll-free area.


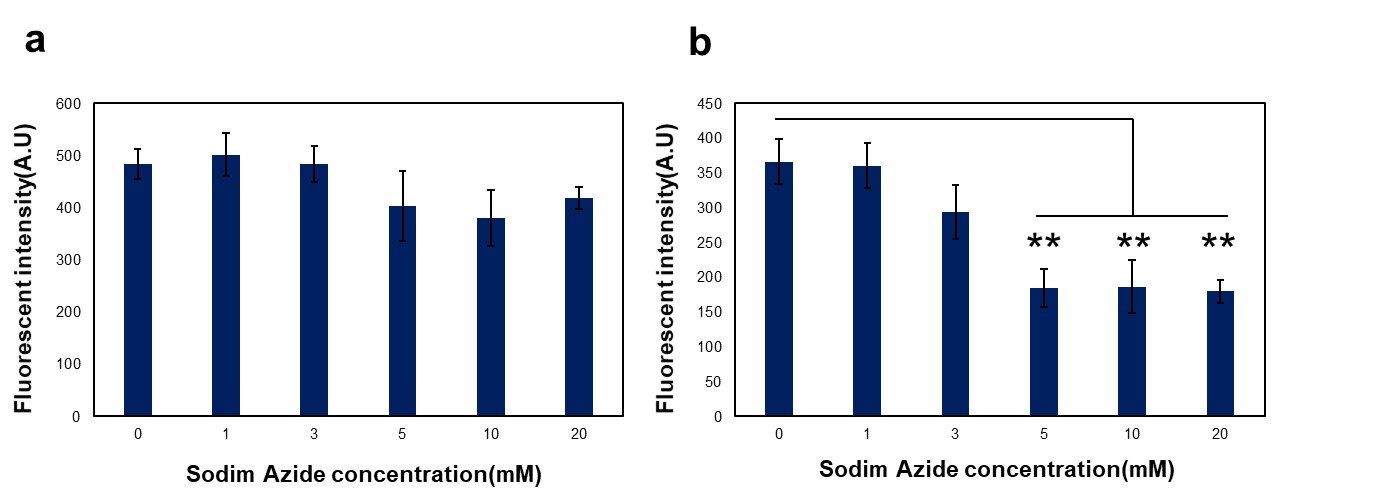


**Figure S2. Inhibition of endocytosis by sodium azide (SA).** (a) Translocation of FITC-pVEC. The fluorescent intensity was measured at 511nm. Sodium azide (SA) was treated for 1 hour with various concentration of SA. The data represent the average of n=5 replicate experiments. Standard deviation bars are shown. No significant difference was observed. (b) Translocation of FITC-Alcohol dehydrogenase by pVEC. $**$ Significantly different (Student’s t-test, p <0.05)
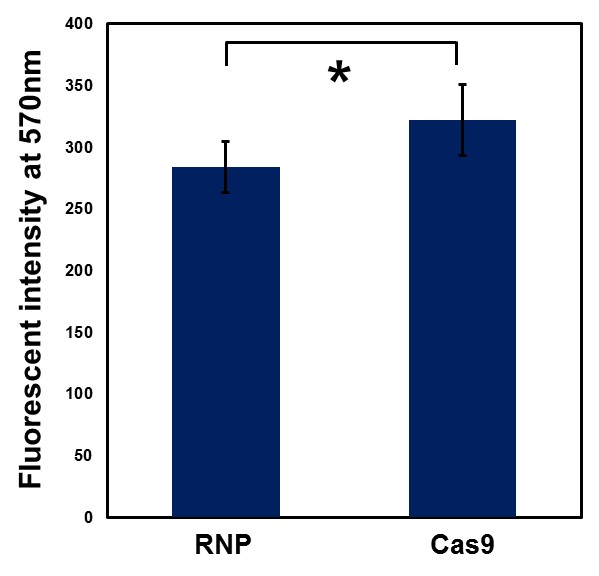


**Figure S3. Translocation of Cas9-cy3.** The washed sample was resuspended in 200ul of TAP media and its fluorescent intensity at 570nm was measured by a spectrofluorophotometer with excitation wavelength of 550nm.
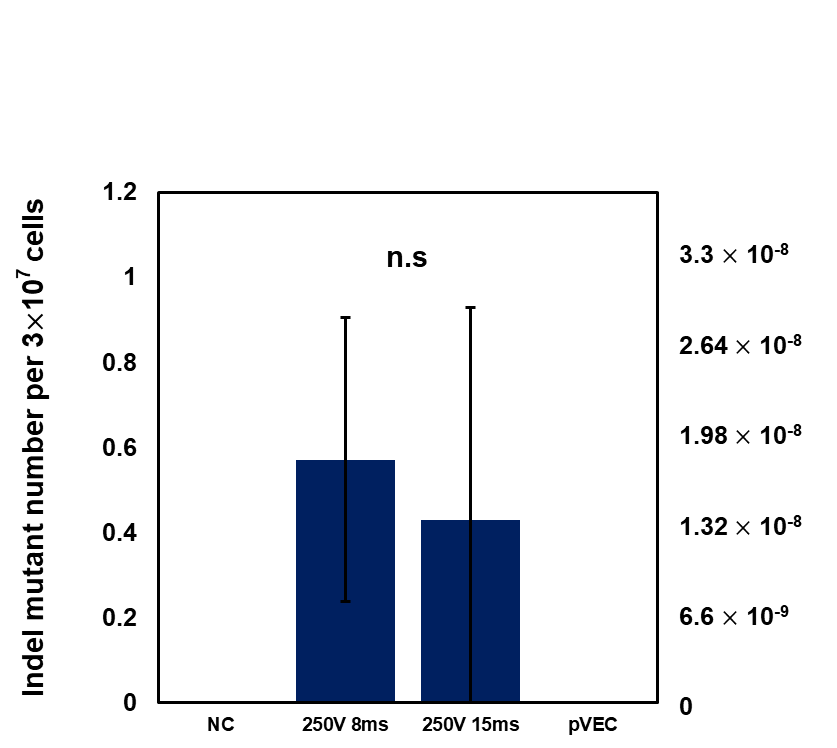


**Figure S4. Electroporation of UVM11 with RNP**  The data represent the average of n=7 replicate experiments. Standard deviation bars are shown. No significant difference was observed.


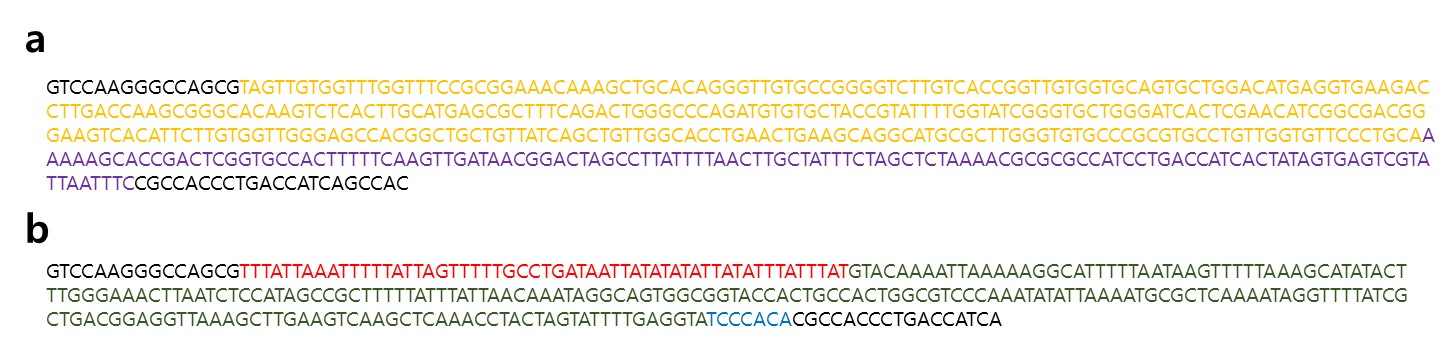


**Figure S5. Other type of insertional mutation in FKB12 gene disruption experiment.** (a) Yellow sequence refers to *Chlamydomonas reinhardtii* predicted protein coding sequence (XM_001700944.1) demonstrated in “The *Chlamydomonas* Genome Reveals the Evolution of Key Animal and Plant Functions“,(Science, 2007, Sabeeha et al). Purple sequence refers *to in-vitro* transcription DNA template. (b) red sequence is unknown, green sequences refers to chloroplast DNA.

|  | **F** | **R** |
| --- | --- | --- |
| ***In-vitro* transcription DNA template** |  | AAAAAAAGCACCGACTCGGTGCCACTTTTTCAAGTTGATAACGGACTAGCCTTATTTTAACTTGCTATTTCTAGCTCTAAAAC |
|  | ***Maa7***  GAAATTAATACGACTCACTATAGCATAGCGACCATTTGCGTCCGTTTTAGAGCTAGAAAT |  |
|  | ***FKB12***  GAAATTAATACGACTCACTATAGTGATGGTCAGGGTGGCGCGCGTTTTAGAGCTAGAAAT |  |
| **PCR amplicon for *in-vitro* cleavage for Maa7** | TCGGTTTGGAGTGGTATGGC | CGACCCACGTAGTCCTTCAG |
| **PCR amplicon for *in-vitro* cleavage for FKB12** | ATGAGCAACTTGGTTCGCAG | TGCGGGGAAAGGCAGTCAAA |
| **FKB12_sequencing1** | AACCTGCCTACCTACCCTCA | CCATACGTTCATACCCCCAGC |
| **FKB12_sequencing2** | GGAAACCTGCACAACAACCT | CCCTGTTGCCCCTAACCG |

Table S1. Primers used in experiments.
